# Two novel blood-based biomarker candidates measuring degradation of tau are associated with dementia: A prospective study

**DOI:** 10.1371/journal.pone.0194802

**Published:** 2018-04-11

**Authors:** Jesper Skov Neergaard, Katrine Dragsbæk, Claus Christiansen, Morten Asser Karsdal, Susanne Brix, Kim Henriksen

**Affiliations:** 1 Nordic Bioscience A/S, Herlev, Denmark; 2 DTU Bioengineering, Technical University of Denmark, Kgs, Lyngby, Denmark; Nathan S Kline Institute, UNITED STATES

## Abstract

**Background:**

Truncated tau appears to be specifically related to disease pathology and recent studies have shown the presence and elevation of several truncated tau species in Cerebrospinal fluid (CSF) of subjects with Alzheimer’s disease (AD); however, the relevance of truncated Tau measurements in blood is still being studied.

**Objective:**

The aim of the current study was to assess the longitudinal associations between baseline levels of two novel blood biomarker candidates measuring truncated tau, Tau-A and Tau-C, and the risk of incident dementia and AD in elderly women.

**Methods:**

Using solid phase competitive ELISA, two tau fragments were detected in serum of 5,309 women from the Prospective Epidemiological Risk Factor study. The study was an observational, prospective study of Danish postmenopausal women. Subjects were followed with registry-linkage for up to 15 years (median follow-up time 13.7 years). Cox regression was used to assess the utility of the biomarker candidates in relation to dementia and AD.

**Results:**

High levels of Tau-A and Tau-C (above the median) in blood were associated with lower risk of dementia and AD (Tau-A: Dementia HR[95% CI] = 0.85[0.70–1.04]; AD 0.71[0.52–0.98] and Tau-C: Dementia 0.84[0.70–1.00]; AD 0.78[0.60–1.03]). Tau-C gave a very modest increase in the AUC in a 5-year prediction horizon as compared to a reference model with age and education, while a combination of the two did not improve their predictive capacity.

**Conclusions:**

Measurement of tau in serum is feasible. The serological tau turnover profile may be related to the diagnosis and development of dementia and AD. The exact processing and profile in serum in relation to cognitive disorders remains to be further assessed to provide simple non-invasive tests to identify subjects with progressive cognitive disorders.

## Introduction

The global burden of dementia is rising, with a new case registered every 3.2 seconds. Dementia is ranked as the 9^th^ most burdensome disease for people aged 60 years and older, however the costs associated with dementia are enormous and place dementia as the most expensive disease in the United States. The reason for this increase in dementia prevalence and the following increased costs are mainly caused by the shifting epidemiological trend of increasing numbers of elder people, caused by low fertility rates and increasing longevity [1,2].

To counteract this dreary trend there is a need for better treatments. The success in pharmaceutical drug development has been greatly challenged due to the difficulties in detecting the disease at a stage allowing for intervention and thereby detecting efficacy. Consequently, there is a clear need for non-invasive and reliable biomarkers to aid in early diagnosis, prognosis and early efficacy assessment. Cerebrospinal fluid (CSF) biomarkers exist, and while they aid in diagnosis, their clinical utility is limited due to the invasive nature of the lumbar puncture.

Evidence suggests that tau is possibly *the* protein triggering and driving the process of cognitive decline and neuronal loss in Alzheimer’s disease (AD)[3,4]. Besides AD, tau is known to be involved in the pathogenesis of several other dementias. The common denominator for these diseases is an alteration of the tau protein leading to the generation of neurotoxic tau species, i.e. neurofibrillary tangles (NFTs) in AD[5]. During this process the tau protein is known to undergo several different posttranslational modifications, where phosphorylation is among the most well studied. Several studies indicate that proteolytic processing of tau plays an important role in neurodegeneration and it has been suggested that caspase cleavage of tau may precede the hyper-phosphorylation, where especially caspase cleavage at Asp421 has been shown to initiate the cascade leading to tau aggregation[6–8].

Recently our research group developed two solid phase competitive ELISA assays detecting the caspase-generated fragment cleaved at Asp421 (Tau-C) and another detecting an ADAM10-generated fragment cleaved at Ala152 (Tau-A) of tau. These novel biomarker candidates have shown promising results in the initial biological validation: In ice hockey players suffering from mild traumatic brain injury, serum levels of Tau-C were significantly higher in post-concussion samples compared with preseason samples[9], confirming that tau processing and release into the circulation is associated with brain damage. Further, levels of Tau-A correlated with the duration of post-concussive symptoms, clearly indicating relevance to the neuronal damage[9]. In a smaller dementia cohort the tau fragments have been shown to be able to discriminate between AD and Mild Cognitive Impairment (MCI) which shows that the tau fragments can provide guidance on the differential diagnosis of dementia[10].

The aim of the current study was to assess the longitudinal associations between baseline levels of Tau-A and Tau-C in serum and the risk of incident dementia and Alzheimer’s disease in a large prospective cohort of 5,309 elderly women.

## Materials and methods

### Study population

The Prospective Epidemiological Risk Factor (PERF) study was an observational, prospective study of Danish postmenopausal women. The cohort has been described in details elsewhere[11]. A total of 5,855 women aged 55–85 were enrolled in the study. Being woman and postmenopausal were the only inclusion criteria’s at the time of enrolment. The baseline examination took place between 1999 and 2001 and comprised a questionnaire, physical examination and blood sampling at the study site. The study was carried out in accordance with ICH-GCP with study protocol approval from The Research Ethics Committee of Copenhagen County. Written informed consent was obtained from all subjects prior to any study related procedures.

Of the entire baseline population (n = 5,855), a total of 206 subjects were excluded based on their cognitive performance at baseline, indicating cognitive impairment consistent with dementia (a Short Blessed Test score ≥10). Two hundred fifty-three subjects did not complete the cognitive testing at baseline and were also excluded in the current study. In addition, 12 subjects were excluded from the analysis due to a preexisting dementia diagnosis derived from the National Danish Patient Registry prior to study enrolment. Further 75 subjects were excluded since no serum samples was available for biomarker measurement. The analytical sample in the current study therefore constituted 5,309 subjects.

### ELISA methodology

The neo-epitope fragments of tau were detected using solid phase competitive ELISA. Fragments were detected by mouse monoclonal antibodies raised against human tau. The antibodies detect an ADAM10-generated cleavage site at Ala152 (Tau-A) and the caspase-3-generated cleavage site at Asp421 (Tau-C). The monoclonal antibodies recognize a decamer sequence containing the cleavage site. Both assays have previously been described in details elsewhere[12,13]. The lower limit of quantification (LLOQ) for Tau-A was 29.4 ng/ml. For Tau-A 68% (n = 3,595) of samples were below the LLOQ. If their reported value were above the lower limit of detection (LLOD, n = 3,443) and their respective Intra-Assay Coefficients of Variability (CV) allowed for it (<15%) these samples were assigned their absolute value (n = 2,293) (see S1 Fig for flow chart on sample inclusion/exclusion). In total 1,150 samples in the range between LLOQ and LLOD were excluded from the main analysis due to an Intra-Assay CV ≥ 15%. A sensitivity analysis including these samples was performed as outlined in the statistical analysis section. The LLOD was 9.3 ng/ml. Samples measured below the LLOD were assigned the LLOD value (n = 152). The LLOQ for Tau-C was determined as 8.6 ng/ml. For Tau-C, samples measured below the LLOQ were assigned the LLOQ value (n = 139). The LLOD for Tau-C was 0.8 ng/ml.

To avoid confounding from plate to plate variation, a series of QC samples were run on 25 plates to provide the range of these measurements with variation. These controls were then included on all plates during the analytical run, and only plates fulfilling the criterion of less than 20% variation from the original validation data set are accepted as valid, and alternatively rerun if this value was too high. The biomarker analysis were conducted at a College of American Pathology (CAP) certified central laboratory (Nordic Bioscience Laboratory). The staff at the central laboratory had no knowledge of the study participants.

### Dementia diagnosis

Follow-up information on dementia status was retrieved from the National Danish Patient Registry and the National Danish Causes of Death Registry using a unique personal identification number for each subject. The follow-up started on the day of study enrollment and ended at the occurrence of an event (dementia diagnosis), death, or on the day of the retrieval of registry data (December 31^th^ 2014), whichever came first. A total of 538 incident dementia cases were identified from the registries. Dementia diagnoses were classified according to The International Classification of Diseases, 10 ^th^ revision (ICD10). The following codes were considered a dementia diagnosis: F00-F04, G30-G32 and R54, while F00 and G30 was used to identify AD (n = 232); however, the registries do not grant access to the data supporting the diagnoses.

### Statistical analysis

Statistical analysis was conducted using R version 3.3.1 (R Foundation for Statistical Computing, Vienna, Austria). Serum levels of Tau-A and Tau-C were log-transformed to account for the skewness and then z-score standardized using the population mean and standard deviation (SD). In cause-specific Cox proportional hazards regression models, all-cause dementia and Alzheimer’s disease were used as the dependent variables. Age was used as timescale and event-free mortality was included as a competing risk as outlined by Benichou and Gail[14]. Levels of the tau fragments were included as either a continuous variable to reflect the risk associated with a change of one SD on the log scale, or as a categorical variable either dichotomized at the median (the group below the median was used as reference) or divided into quartiles (the lowest quartile (Q1) was used as reference). Initially we modeled the crude risk in the separate univariate analysis (model 1). Secondly, in addition to age, we adjusted for education level (primary school, high school and university) (model 2). Lastly we made a multivariate model adjusted for the continuous variables; age (as timescale), body mass index (kg/m^2^), platelet count (10^9^/L), white blood cell count (10^9^/L), albumin (mmol/L), alkaline phosphatase (unit/L), gamma glutamyltransferase (unit/L), high-density lipoprotein (mmol/L) and the categorical variables; education level, smoking (never, past or current), alcohol consumption (never, <10.5 alcohol units/week, 10.5–21 alcohol units/week or >21 alcohol units/week), physical activity (other than walking) (never, once weekly, twice weekly or three or more times per week) (model 3). Selection of covariates was based on significant association with levels of Tau-A and Tau-C using a multiple linear regression analysis (data not shown) and relevant risk factors as reported in the literature. A sensitivity analysis was performed for Tau-A by including all samples between LLOQ and LLOD with an intra-assay CVs above the initial requirement of <15%.

The cumulative incidence of a dementia event in a competing risk framework taking the risk of death without dementia into account was illustrated in quartiles of Tau-A and Tau-C. The cumulative incidence was estimated using the Aalen–Johansen method[15]. The difference between cumulative incidence curves was tested using the modified χ^2^ statistic outlined by Gray[16].

Finally we investigated the predictive value of the two biomarker candidates when added *i)* to a reference model containing age and educational level and *ii)* to a reference model containing all the independent variables from the multivariate model. The predictive value was assessed by computing the area under the Receiver-Operating Characteristics curve (AUC) for a 5-year and 10-year prediction horizon for each of the individual markers and their combination, using time from baseline as timescale.

## Results

Selected baseline characteristics of the study population are summarized in Table 1. During the follow-up period of maximum 15 years (median follow-up time 13.7 years) a total of 538 incident dementia cases were identified from the registries, of which 232 had AD.

**Table 1:** Study population characteristics at baseline. Numbers are shown as absolute numbers with percentile in brackets for categorical variables. For numerical variables, the mean (standard deviation) is shown.

Parameter	Dementia (n = 538)	Controls (n = 4771)
**Age, mean (SD) (years)**	70.0 (6.4)	75.1 (5.4)
**Highest level of education**		
Primary school, n (%)	386 (72)	3357 (70)
High School, n (%)	107 (20)	1061 (22)
University, n (%)	44 (8)	347 (7)
**BMI, mean (SD) (kg/m**^**2**^**)**	26.2 (4.2)	25.8 (4.2)
**Smoking**		
Never, n (%)	260 (48)	2272 (48)
Past, n (%)	180 (33)	1428 (30)
Current, n (%)	98 (18)	1067 (22)
**Alcohol**		
Never, n (%)	250 (47)	2012 (42)
<10.5 units/week, n (%)	126 (24)	1126 (24)
10.5–21 units/week, n (%)	128 (24)	1256 (27)
>21 units/week, n (%)	32 (6)	341 (7)
**Physical activity**		
Never, n (%)	195 (36)	1398 (29)
1 time/week, n (%)	112 (21)	1015 (21)
2 times/week, n (%)	70 (13)	632 (13)
3+ times/week, n (%)	159 (30)	1722 (36)
**Hypertension, n (%)**	182 (34)	1435 (30)
**History of stroke, n (%)**	24 (4)	133 (3)
**Diabetes, n (%)**	11 (2)	135 (3)
**Depression/Anxiety, n(%)**	50 (9)	295 (6)
**Tau-A level, mean (SD) (ng/mL)**	28.3 (16.0)	26.8 (14.5)
**Tau-C level, mean (SD) (ng/mL)**	22.0 (11.9)	20.3 (10.4)

Cause-specific Cox proportional hazards regression models were used to assess the association between the biomarker levels and the risk of incident dementia or AD as listed in Table 2. Higher levels of Tau-C, both as a continuous measure and categorized, were associated with a decreased risk of all-cause dementia and AD in the age-adjusted model. Subjects in the highest quartile had a 29% (HR [95% CI] 0.71 [0.55–0.91]) decreased risk of dementia and a 34% (HR [95% CI] 0.66 [0.46–0.96]) decreased risk of AD as compared to subjects within the lowest quartile. A dose-response tendency was observed for Tau-C in all three models, indicating decreasing risk of dementia with increasing levels of the biomarker.

**Table 2:** Association of Tau-A and Tau-C with the risk of incident dementia and Alzheimer’s disease.

			Continuous (per log SD decrease)			Dichotomized at median			Quartiles									
						> (Ref: <)			Q1	Q2			Q3			Q4		
Biomarker		Outcome	HR	95% CI	P	HR	95% CI	P	HR	HR	95% CI	P	HR	95% CI	P	HR	95% CI	P
M1	Tau-A	Dementia	0.87	0.79–0.96	**0.005**	0.81	0.67–0.98	**0.03**	Ref	0.94	0.72–1.22	0.6	0.83	0.63–1.09	0.2	0.74	0.57–0.98	**0.03**
		AD	0.83	0.72–0.96	**0.01**	0.68	0.50–0.91	**0.01**	Ref	0.82	0.56–1.20	0.3	0.63	0.41–0.95	**0.03**	0.61	0.40–0.91	**0.02**
	Tau-C	Dementia	0.88	0.80–0.96	**0.005**	0.79	0.67–0.94	**0.007**	Ref	0.89	0.71–1.11	0.3	0.80	0.64–1.01	0.06	0.71	0.55–0.91	**0.006**
		AD	0.83	0.72–0.95	**0.009**	0.71	0.55–0.93	**0.01**	Ref	0.84	0.60–1.18	0.3	0.66	0.46–0.94	**0.02**	0.66	0.46–0.96	**0.03**
M2	Tau-A	Dementia	0.87	0.79–0.96	**0.004**	0.81	0.67–0.98	**0.03**	Ref	0.94	0.72–1.22	0.6	0.83	0.63–1.09	0.2	0.74	0.57–0.98	**0.03**
		AD	0.83	0.72–0.96	**0.01**	0.68	0.50–0.91	**0.01**	Ref	0.82	0.56–1.21	0.3	0.63	0.41–0.95	**0.03**	0.61	0.40–0.91	**0.02**
	Tau-C	Dementia	0.88	0.80–0.96	**0.006**	0.79	0.67–0.94	**0.008**	Ref	0.89	0.71–1.12	0.3	0.81	0.64–1.02	0.07	0.71	0.56–0.91	**0.008**
		AD	0.83	0.72–0.95	**0.008**	0.71	0.55–0.93	**0.01**	Ref	0.84	0.60–1.17	0.3	0.66	0.46–0.94	**0.02**	0.66	0.46–0.96	**0.03**
M3	Tau-A	Dementia	0.89	0.81–0.99	**0.03**	0.85	0.70–1.04	0.1	Ref	0.98	0.75–1.28	0.9	0.88	0.66–1.16	0.4	0.79	0.59–1.06	0.1
		AD	0.87	0.74–1.01	0.07	0.71	0.52–0.98	**0.03**	Ref	0.89	0.60–1.32	0.6	0.67	0.44–1.04	0.07	0.67	0.43–1.04	0.07
	Tau-C	Dementia	0.90	0.82–0.99	**0.03**	0.84	0.70–1.00	**0.05**	Ref	0.89	0.71–1.12	0.3	0.84	0.66–1.06	0.1	0.76	0.58–0.98	**0.03**
		AD	0.87	0.75–1.00	**0.05**	0.78	0.60–1.03	0.08	Ref	0.87	0.62–1.23	0.4	0.72	0.50–1.05	0.09	0.75	0.51–1.11	0.1

Ref: reference group. M1: Model 1. This model was adjusted for age. M2: Model 2. This model was adjusted for age and education. M3: Model 3. This model was adjusted for: age, education level, body mass index, smoking (never, past or current), alcohol consumption (never, <10.5 alcohol units/week, 10.5–21 alcohol units/week or >21 alcohol units/week), physical activity (other than walking) (never, once weekly, twice weekly or three or more times per week) platelet count, white blood cell count, albumin (mmol/L), alkaline phosphatase (unit/L), gamma glutamyltransferase (unit/L), high-density lipoprotein (mmol/L). P≤0.05 are marked with bold.

The association between Tau-C and incident dementia and AD remained significant after adjustment for age and education and in the multi adjusted model. Tau-C levels in the highest quartile were associated with a 29% lower risk of dementia and 34% lower risk of AD, when adjusted for age and education. In the multi adjusted model, subjects in the highest quartile had a 24% (HR [95% CI] 0.76 [0.58–0.98]) lower risk of dementia as compared to subjects in the lowest quartile. Further, the risk of dementia and AD decreased 10% (HR [95% CI] 0.90 [0.82–0.99]) and 13% (HR [95% CI] 0.87 [0.75–1.00]) with every log SD increase of the biomarker, respectively.

A dose-response relation was also observed with across the quartiles of Tau-A, and as continuous measure the risk decreased 13% (HR [95% CI] 0.87 [0.79–0.96]) in relation to all-cause dementia and 17% (HR [95% CI] 0.83 [0.72–0.96]) in relation to AD with every log SD increase of the biomarker in the age and educationally adjusted model, respectively. When dichotomized at the median subjects above the median had a 19% lower risk of dementia (HR [95% CI] 0.81 [0.67–0.98]) and a 32% lower risk of AD (HR [95% CI] 0.68 [0.50–0.91]). After multi factor adjustment the association between Tau-A and incident dementia and AD vanished, however the association remained significant between Tau-A and all-cause dementia as a continuous measure and in the dichotomized analysis, where subjects above the median had 29% decreased risk of AD (HR [95% CI] 0.71 [0.52–0.98]). The sensitivity analysis for Tau-A did not alter the overall results. Thus, there was a minor tendency for both outcomes where the HRs was shifted modestly towards the null (data not shown).

**Fig 1:**
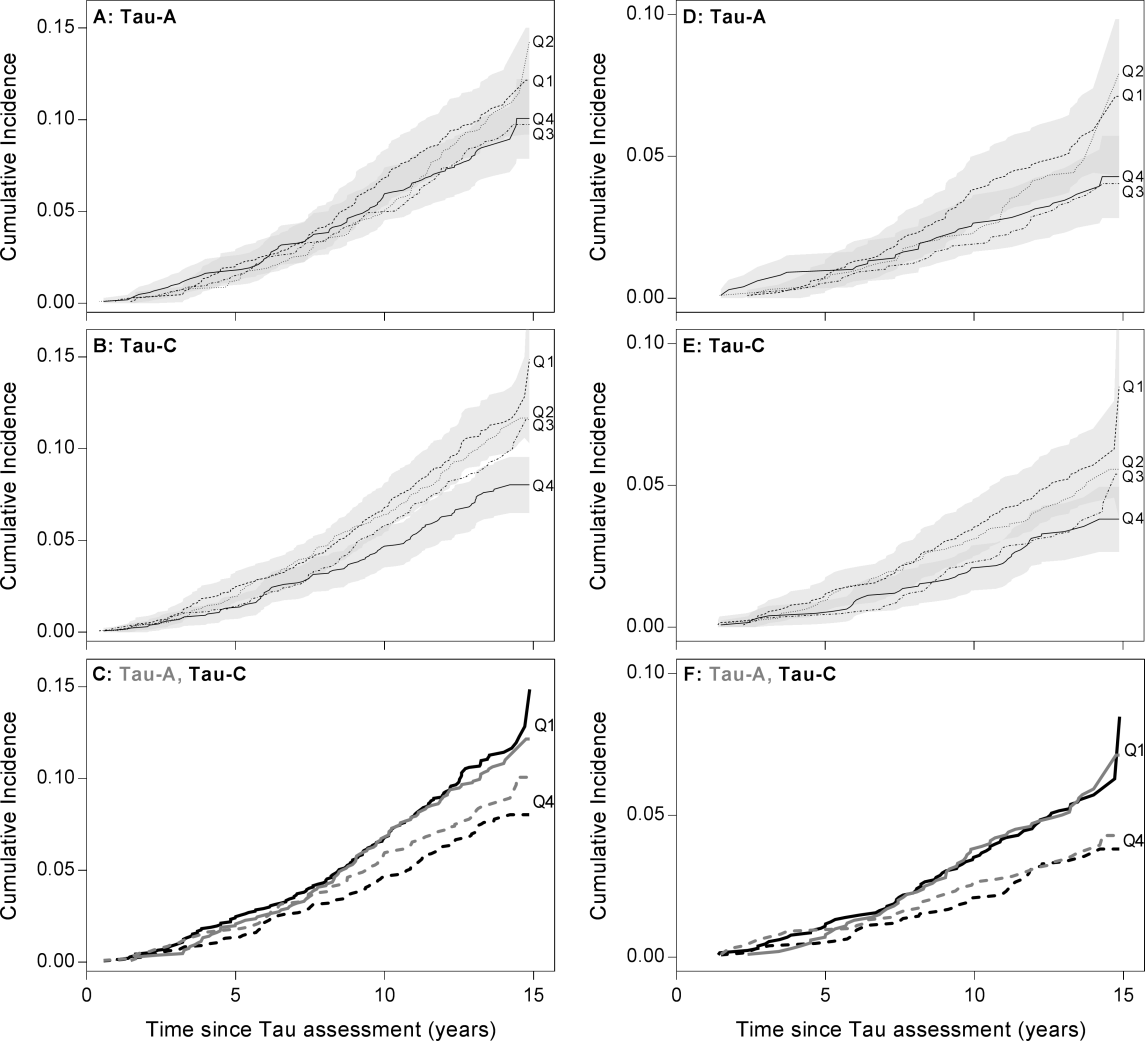
Cumulative incidence curves. Cumulative incidence in quartiles of Tau-A and Tau-C as a function of follow-up time. Left panel (A-C) illustrates the cumulative incidence for all-cause dementia. Right panel (D-F) illustrates the cumulative incidence for AD. The bottom graphs (C and F) are overlay plots of Tau-A and Tau-C showing only Q1 and Q4 for both markers. Confidence intervals (95%) are shown for the lowest (Q1) and highest quartiles (Q4) in A, B, D and E.

The two tau biomarker candidates were also stratified into quartiles and illustrated as cumulative incidence curves (Fig 1).

The analysis showed that the separation between Q1 and Q4 for Tau-A in relation to all-cause dementia is poor, and with significant overlap between confidence intervals for Q1 and Q4 (Fig 1A, p = 0.2). For AD on the other hand, the separation between Q1 and Q4 is larger (Fig 1D, p = 0.03). For Tau-C, the separation between the quartiles is larger and with only minor overlap between the confidence limits of Q1 and Q4 (Fig 1B, E, p = 0.0009 for dementia and p = 0.01 for AD). Moreover, a dose-response relation across the four quartiles is observed for Tau-C in relation to both dementia and AD. The overlay plots (Fig 1C and 1F) illustrate Q1 and Q4 for both biomarkers. It appears that the distance and thereby the separation between Q1 and Q4 increases from Tau-A to Tau-C.

As outlined in Table 3, Tau-A did not improve the risk prediction of dementia or AD within a 5-year and 10-year prediction horizon. Using a 5-year prediction horizon, Tau-C improved the prediction for both dementia and AD minimally, with an AUC change of 0.01 and 0.02, respectively (p = 0.05 for both), although only with model 2 as the reference model. In the 10-year prediction horizon Tau-C did not improve the prediction as compared to any of the reference models. A combined model including both Tau-A and Tau-C did not improve their predictive capacity.

**Table 3:** Improvement in risk prediction with the addition of Tau-A, Tau-C or Tau-A and Tau-C.

			Model 2: Age + Education			Model 3: Multivariate		
Prediction horizon	Outcome	Model	AUC	(95% CI)	P*	AUC	(95% CI)	P*
within 5 years	Dementia	Reference	0.77	(0.72–0.82)		0.79	(0.74–0.84)	
		+ Tau-A	0.77	(0.73–0.82)	0.9	0.79	(0.74–0.84)	0.7
		Reference	0.76	(0.72–0.80)		0.78	(0.74–0.82)	
		+ Tau-C	0.77	(0.72–0.81)	**0.05**	0.78	(0.74–0.83)	0.2
		Reference	0.77	(0.72–0.82)		0.79	(0.74–0.84)	
		+ Tau-A +Tau-C	0.78	(0.73–0.83)	0.2	0.79	(0.74–0.84)	0.9
	AD	Reference	0.80	(0.73–0.86)		0.82	(0.75–0.89)	
		+ Tau-A	0.79	(0.72–0.85)	0.4	0.81	(0.73–0.88)	0.3
		Reference	0.78	(0.71–0.84)		0.82	(0.75–0.89)	
		+ Tau-C	0.80	(0.74–0.86)	**0.05**	0.83	(0.76–0.89)	0.2
		Reference	0.80	(0.73–0.86)		0.82	(0.75–0.89)	
		+ Tau-A +Tau-C	0.80	(0.74–0.87)	0.6	0.82	(0.75–0.89)	0.96
within 10 years	Dementia	Reference	0.77	(0.74–0.80)		0.77	(0.75–0.80)	
		+ Tau-A	0.77	(0.74–0.80)	0.4	0.78	(0.75–0.80)	0.6
		Reference	0.76	(0.74–0.79)		0.77	(0.74–0.79)	
		+ Tau-C	0.76	(0.74–0.79)	0.7	0.77	(0.74–0.79)	0.97
		Reference	0.77	(0.74–0.80)		0.77	(0.75–0.80)	
		+ Tau-A +Tau-C	0.77	(0.74–0.80)	0.5	0.78	(0.75–0.80)	0.6
	AD	Reference	0.76	(0.72–0.80)		0.77	(0.73–0.81)	
		+ Tau-A	0.77	(0.73–0.80)	0.5	0.78	(0.74–0.81)	0.6
		Reference	0.74	(0.71–0.77)		0.75	(0.72–0.79)	
		+ Tau-C	0.75	(0.72–0.78)	0.1	0.76	(0.72–0.79)	0.3
		Reference	0.76	(0.72–0.80)		0.77	(0.73–0.81)	
		+ Tau-A +Tau-C	0.77	(0.73–0.81)	0.2	0.78	(0.74–0.82)	0.3

*Compared with reference model, P≤0.05 are marked with bold

## Discussion

In this study, we assessed the prognostic utility of two novel serum biomarker candidates of neurodegeneration in a large prospective study. Both biomarkers, Tau-C in particular, were associated with incident dementia, where high levels of the biomarkers were associated with lower risk of incident dementia and AD.

The inverse association between levels of tau and risk of dementia seems counterintuitive since higher levels of tau are found in the CSF of subjects with AD and to a lesser degree in other types of dementia[17–19]. Sparks and colleagues, however, also found lower levels of tau in plasma of AD patients and explain their association with a reduced transport of excess central tau to the periphery, caused by pathological alterations of tau[20]. Likewise, a similar situation has been observed with the Glial Cell-Line Derived Neurotrophic Factor protein in AD subjects. Here, the protein level is decreased in serum and increased in CSF in AD versus control subjects. The authors speculate that it could be related to an altered function of the blood-brain barrier thus disturbing clearance or facilitating crossing of potentially harmful fragments in the healthy brain[21]. Another plausible explanation is linked to neuroinflammation where microglia exhibit significant phenotypic changes during the course of the disease. In early AD microglial activation is believed to be neuroprotective by enhancing phagocytosis and degradation of β-amyloid and tau[22,23], a process that may result in less release of tau to the periphery. In later stages, where microglia become over-activated, they lose their phagocytic abilities resulting in uncontrolled inflammation[24]. This would result in higher levels of both central and peripheral tau.

There are previous reports of measurements of tau protein in circulating blood, but most studies are small in size, low in numbers, and show inconsistent results[20,25–31]. A recent meta-analysis has therefore concluded that plasma tau is not a useful marker for AD[32]. The meta-analysis included six studies, whereof some reported an increase[26,28,29], others a decrease[20,25] and one study reported no change in AD patients as compared to healthy age-matched controls[27]. The heterogeneity across the studies illustrates one of the challenges of biomarker assessment in blood. An important difference between the previous studies and ours is that previous studies measured total tau and not truncated tau. Truncated tau appears to be specifically related to disease pathology and recent studies have shown the presence and elevation of several truncated tau species in CSF of AD patients[33]. Besides being more specific for pathological changes than the intact proteins, the truncated fragments might more easily pass through the blood-brain barrier, due to their smaller size, as larger fragments do not cross the barrier.

Another important difference is the setting in which the markers are assessed. With one exception[31], the previous studies of plasma tau are cross-sectional, while ours is longitudinal, and thereby the first large cohort study to assess the prognostic utility of truncated tau in serum. Importantly the longitudinal design limit the concern of reverse causation. Mattson and colleagues recently touched upon the prognostic potential of plasma tau where they found that higher plasma tau was associated with progression, measured as the change in cognitive performance over time, however this was assessed in subjects with MCI and established dementia and not cognitively normal individuals [31]. The biomarker dynamics of Tau-A and Tau-C as a function of disease severity are still to be elucidated, but based on the current observations we speculate that the levels of the biomarkers are time-dependent and may change direction during the course of the disease. In minor cross-sectional studies of dementia and mild traumatic brain injury subjects, we found that the levels of Tau-A and Tau-C were elevated in diseased versus control subjects[9,10]. Associations with opposite direction as to what we found in this prognostic analysis. While the influence on the disease path after processing of tau by ADAM10 is unknown, evidence suggests that the caspase cleavage leading to the generation of Tau-C may play an important role in the cascade leading to tau aggregation[34]. The Tau-C fragment has been found to be one of the truncated tau forms in NFTs[8]. This evidence suggests that Tau-C may accumulate within the neurons during the process of NFT formation, and eventually be released to the circulation, at a more advanced disease stage, where the NFT load is sufficient to cause neuronal cell death. This process could explain the associations we have observed in our cross-sectional and longitudinal studies, respectively. There are previous indications of a non-linear relation between tau and disease severity over time. Using data from the ADNI database, Mouiha and colleagues investigated the time course of the CSF biomarkers, Aβ, t-tau and p-tau, and for all three markers the most likely model describing the relation between the biomarkers and disease severity was non-linear[35]. The most likely time course for t-tau and p-tau was found to be a penalized B-spline model, where multiple inflexion points could indicate multiple phases of accumulation as opposed to a continuous, uninterrupted process. Recent longitudinal data from the DIAN study also suggest that the biomarker trajectories may differ as a function of disease severity[36].

While the associations of Tau-C and Tau-A with incident dementia and AD revealed a potential value of these novel biomarker candidates, their predictive value as individual markers was limited. Tau-C gave a very modest increase in the AUC in a 5-year prediction horizon as compared to a reference model based on age and education, however the increase in AUC vanished in the fully adjusted model. It must be noted that this assessment was done in a population-based cohort without any specific enrichment e.g. a requirement for Aβ positivity. The heterogeneous population may leach out the predictive performance. Despite the limited predictive value as stand-alone biomarkers, it is likely that the markers could be useful in combination with other serum biomarkers e.g. other tau-species and β-amyloid. From our study it is clear that the interpretation of a peripheral signal and its relation to alterations within the brain is difficult, albeit studies, including the current study, begin to highlight that serological assessment of pathophysiological tau processing is possible. Understanding the link between the biomarker signal and the pathophysiological processes is however of paramount importance and studies that can reveal the time-dependency and biomarker dynamics in relation to disease severity e.g. with repeated measures should therefore be a priority for the future.

A peripheral biomarker of biologically processed tau has several advantages as compared to β-amyloid. First, while the Amyloid Precursor protein is also expressed in peripheral tissues like the pancreas, kidney, heart and liver[37], animal studies suggest that circulating tau protein arises from central neurons[38]. This implies that the peripheral pool of tau would arise directly from the brain, while β-amyloid in plasma or serum probably reflects a mixture of peripheral and brain-derived protein. This might make the interpretation of a peripheral tau signal easier, although the processing, release and transport of tau from the brain to the periphery is yet not fully understood. Similar to a previous study of total tau measured in plasma, our markers did not show any correlation with t-tau or p-tau levels in CSF suggesting that the steady-state concentrations of tau are differentially regulated in these two body fluids[10,26]. Secondly, it has become quite clear that, although CSF Aβ aids in the early diagnosis of AD, the marker is not related to disease severity and duration[39,40]. CSF Tau, on the other hand, correlates with disease severity during the whole time course of AD[40–42]. An association that is also likely with truncated tau in the periphery. Finally, tau outperformed Aβ in a head-to-head comparison from a recent meta-analysis, where tau proved to have a larger effect size (measured by the disease to control ratio) in both CSF and plasma/serum[43].

### Limitations

Generally the dementia field is hampered by misdiagnosis and underdiagnoses which complicate the evaluation of new diagnostic and prognostic biomarkers. In the current study we used registry-linkage to collect information on dementia diagnoses. This method has the advantage of a very limited loss to follow up, however one could question the validity of the diagnoses due to its origin, and access to specific information regarding this cannot be obtained. Similar registries are found in other countries in Scandinavia, and studies from Sweden and Finland have shown that the diagnoses in the registries have very good accuracy, but underestimation is present. This underestimation may result in an underestimation of the biomarker potential. [44,45]

Like most other studies we based our biomarker assessment on a binary distinction between cases and controls. Since dementia evolves over decades with a long preclinical phase the binary distinction is probably not the most appropriate method as the control group may contain several subjects with preclinical disease at the time of biomarker assessment. Although this is difficult to work around, a long follow-up time as in the current study, is one of the best possibilities to avoid misclassifications.

In literature, variability in assays and detection challenges are reported as two major hurdles with peripheral biomarkers that should be overcome before the full potential of these biomarkers is expressed[46,47]. At least for our Tau-A assay we also faced a challenge with sensitivity, which we hope to overcome with assay optimization. In a sensitivity analysis, we did not observed any significant impact on the overall findings of the samples measured below the LLOQ with intra-assay CVs above 15%. The current study was limited to women and therefore generalization cannot be made to men of same age. The biomarker candidates should be tested in other cohorts to ensure reproducibility and generalizability.

Finally, tau pathology is not specific to AD, but present in multiple dementias[5], a finding the corresponds to our findings which are consistent across dementia forms.

## Conclusions

The current study demonstrates that serological assessment of pathophysiological tau processing is possible. Tau-A and Tau-C measured in serum could be useful prognostic biomarkers to aid in early diagnosis of preclinical dementia and AD. Additional validation in relation to prognosis and time-dependency of these novel biomarkers should be a subject for future investigations.

## Supporting information

S1 Fig Flow chart on sample inclusion and exclusion. (TIF)

S1 File Dataset with data used for the analysis. (TXT)
