# Supplementary figures and images for: Two novel blood-based biomarker candidates measuring degradation of tau are associated with dementia: A prospective study
Source: PLoS One. 2018 Apr 11;13(4):e0194802. doi: 10.1371/journal.pone.0194802 (PMC5895005; doi:10.1371/journal.pone.0194802)

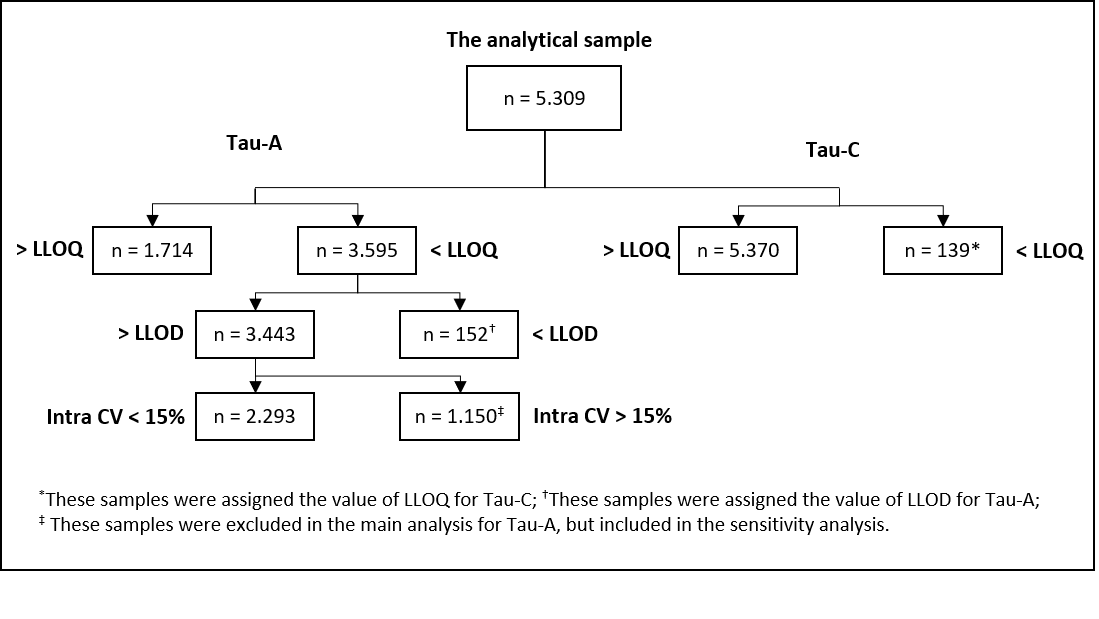

Supplement: S1 Fig — (TIF) [file pone.0194802.s001.tif]
